# Endoscopic Submucosal Single- or Multi-tunnel Dissection for Near-Circumferential and Circumferential Superficial Esophageal Neoplastic Lesions

**DOI:** 10.1155/2019/2943232

**Published:** 2019-03-12

**Authors:** Lin-Lin Zhu, Jun-Chao Wu, Yi-Ping Wang, Du He, Wen-Yan Zhang, Tao Gan, Jin-Lin Yang

**Affiliations:** ^1^Department of International Medical Center, West China Hospital of Sichuan University, No. 37 Guo Xue Alley, Chengdu, 610041 Sichuan, China; ^2^Department of Gastroenterology and Hepatology, West China Hospital of Sichuan University, No. 37 Guo Xue Alley, Chengdu, 610041 Sichuan, China; ^3^Department of Pathology, West China Hospital of Sichuan University, No. 37 Guo Xue Alley, Chengdu, 610041 Sichuan, China

## Abstract

This study reports the outcomes of endoscopic submucosal single-tunnel dissection or endoscopic submucosal multi-tunnel dissection for the treatment of esophageal neoplastic lesions of at least three-quarters of the esophageal circumference, including circumferential superficial esophageal neoplastic lesions. From July 2014 to February 2018, a total of 124 lesions underwent endoscopic submucosal tunnel dissection at our hospital. One to four submucosal tunnels were created in the oral to anal direction. Of the 124 lesions, there were 83 noncomplete circumferential lesions and 41 circumferential lesions. Endoscopic submucosal single-tunnel dissection was performed in 54 patients, two-tunnel dissection in 43 patients, three-tunnel dissection in 19 patients, and four-tunnel dissection in 8 patients. The mean dissection speed was 22.8 ± 12.7 mm^2^/min. En bloc dissection was achieved in all lesions, and the R0 resection rate was 70.2 percent. No matter how large the lesion area was, there were no significant differences in the dissection speed and the R0 resection rate when lesions were at least three-quarters of the esophageal circumference. Esophageal stricture was observed in 54 patients and was relieved by placement of a retrievable metal stent or by endoscopic water balloon dilation. No recurrence was noted after 19.1 ± 12.4 months of follow-up. Our large sample size study showed that endoscopic submucosal tunnel dissection showed effectiveness and safety for the treatment of large superficial esophageal neoplastic lesions at least three-quarters of the esophageal circumference, including circumferential superficial esophageal neoplastic lesions.

## 1. Introduction

Endoscopic submucosal dissection (ESD) is recommended as the preferred treatment choice for early esophageal squamous cell carcinoma, adenocarcinoma, and precancerous lesions by the Chinese Society of Digestive Endoscopy (CSDE) [1], the Japan Esophageal Society (JES) [2], and the National Comprehensive Cancer Network (NCCN) guidelines. However, the less obvious lifting effect of the submucosal injection and the collapse of the submucosal space have restricted its application in large superficial esophageal neoplastic lesions. To combat these limitations of ESD, Linghu et al. described the use of endoscopic submucosal tunnel dissection (ESTD) [3, 4]. The tunnel between the mucosa and muscularis propria is the core characteristic of ESTD, which is a time-saving and efficient dissection technique for large esophageal neoplastic lesions that is associated with fewer complications [4]. However, some difficulties remain in the use of the single-tunnel ESTD (ESSTD) procedure for the dissection of near-circumferential or circumferential esophageal lesions. The dissection in the desired plane is difficult because of the larger and/or wider tunnel cavity and the collapse of the submucosal space between the mucosa and the muscularis propria, making visualization difficult [5].

Our previous article suggested that endoscopic submucosal multi-tunnel dissection (ESMTD) could be used for the dissection of circumferential esophageal lesions [5]. Using this technique, two or more tunnels are created between the mucosa and muscularis propria, and the submucosal tunnels facilitate endoscopic dissection of lesions. We hypothesized that if the diameter of each tunnel is smaller, the transparent cap held up the walls between the tunnels more efficiently, resulting in a faster wall incision. In addition, it was easier to identify small arterioles in the submucosa and prevent bleeding by electric coagulation due to the clearer field of view. Favorable results have been achieved from 7 patients with circumferential superficial esophageal neoplastic lesions [5].

There are few reports, all with a small sample size, on the use of ESSTD or ESMTD for the treatment of esophageal neoplastic lesions. To provide more evidence regarding the outcomes of ESSTD and ESMTD in treating near-circumferential and circumferential esophageal lesions, we report 124 sequential cases of large esophageal neoplastic lesions that were at least three-quarters of the esophageal circumference.

## 2. Materials and Methods

From July 2014 to February 2018, 124 consecutive patients (aged 41-83 years; 76 males and 48 females) underwent ESSTD or ESMTD at our hospital. Esophageal neoplastic lesions involve at least three-quarters of the esophageal circumference. Written informed consent was obtained from all patients before the operation. This research was reviewed and approved by the Ethics Committee of the West China Hospital of Sichuan University. The inclusion criteria were as follows: (1) lesions involving at least three-quarters of the esophageal circumference, (2) post-ESTD pathological diagnosis of esophageal cancer or precancerous lesions, (3) no evidence of lymph node or distant metastasis, and (4) no prior treatment of chemotherapy or radiation.

### 2.1. Endoscopic Treatments

Endoscopic ultrasonography (EUS) (Pentax EG-3830UT, Pentax EG-3630UR, Pentax, Japan) was performed to evaluate the depth of the lesions in all patients. Magnifying endoscopy with narrow-band imaging (ME-NBI) was used to assess the intrapapillary capillary loops (IPCL) and the lateral spread of the partial lesions before the ESSTD/ESMTD procedure. Contrast-enhanced computed tomography (CT) of the chest and abdomen was carried out to identify lymph node metastasis. A chromoendoscopy was carried out after direct spraying of 15-20 mL of 1.5% iodine solution through the biopsy channel of the endoscope before the endoscopic dissection procedure, a pink color sign was utilized to determine tumor borders.

### 2.2. ESSTD or ESMTD Procedures

Three principal endoscopists (Jin-Lin Yang, Jun-Chao Wu, and Tao Gan) that performed this technique had 15-25 years of experience as endoscopists. An endoscope with a water jet system (GIF-Q260J, Olympus, Tokyo, Japan) was used for the ESTD procedure and had a transparent cap (D-201-10704, Olympus, Tokyo, Japan) attached to its tip. An IT knife (KD-611L, Olympus, Tokyo, Japan), a Dual-Knife (KD-650L/Q, Olympus, Tokyo, Japan), an injection needle (NM-200U-0423, Olympus, Tokyo, Japan), and hemostatic forceps (FD-410 LR, Olympus, Tokyo, Japan) were used during the procedure. The VIO200D and APC-ICC200 (Erbe Elektromedizin GmbH, Germany) were set to the forced coagulation mode (effect 2, output 45 W) to incise the mucosal layer.

**Figure 1:**
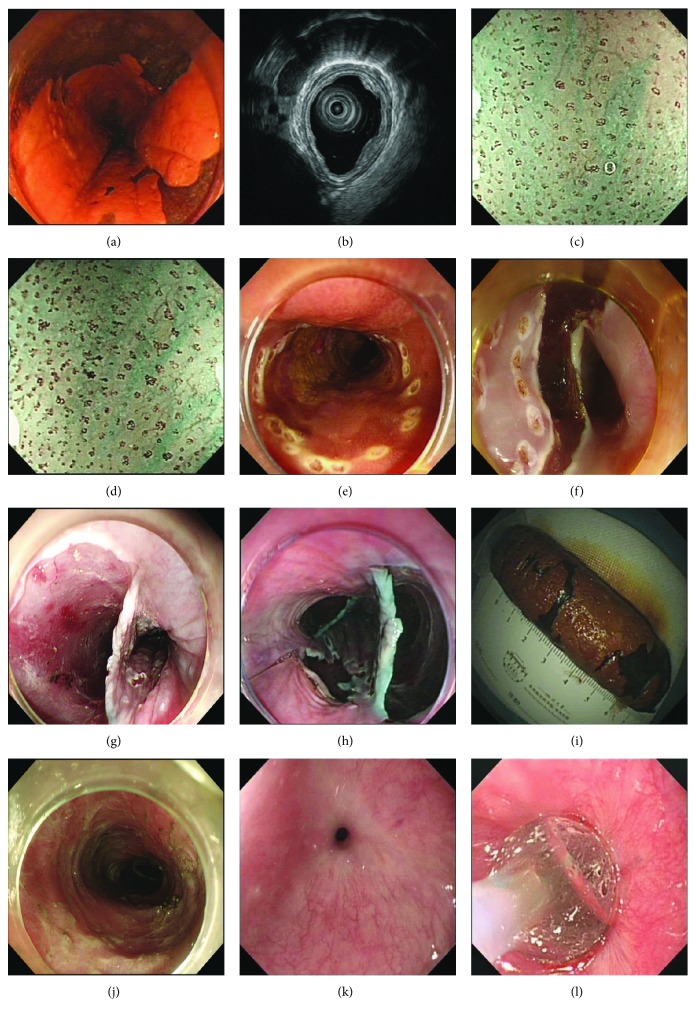
Endoscopic submucosal single-tunnel dissection (ESSTD) or endoscopic submucosal multitunnel dissection (ESMTD) for near-circumferential esophageal lesions. (a) Iodine staining of the lesions. (b) Endoscopic ultrasound (EUS) imaging. (c, d) Magnifying endoscopy with narrow-band imaging (ME-NBI). (e) Marking the margin of the lesion with a Dual-Knife. (f) Posterior tunnel entrance creation by submucosal dissection with an IT knife. (g) Anterior incision after submucosal injection with a Dual-Knife. (h) Operation in three tunnels. (i) The resected posterior lesion specimen cut and flattened on a foam board after the operation. (j) The artificial wound after endoscopic submucosal tunnel dissection (ESTD). (k) Esophageal stricture. (l) Endoscopic water balloon dilation.

The standard ESSTD or ESMTD procedures for near-circumferential esophageal lesions were performed as previously reported [5]. During the ESMTD procedure, the multiple oral-side mucosal dissection served as the last step of the ESMTD procedure (Figure 1) [5].

### 2.3. Complications

Bleeding related to the procedure was defined as requiring postoperative hemostatic treatment, such as endoscopic clipping or thermocoagulation. Perforation was diagnosed when the mechanical damage exceeded the muscularis propria or when mediastinal connective tissue was observed during the procedure. Mediastinal emphysema was diagnosed by the presence of air in the mediastinal space on plain radiography or subcutaneous emphysema of the neck or chest. Postoperative esophageal stricture was present when the gastroscope (diameter: 9.2 mm) could not be successfully passed. The management of esophageal stricture in all patients is mainly due to composite factors from both patients and physicians. (1) Clinicians advised the alternative treatment for postoperative esophageal stricture according to the severity of esophageal stenosis (Stooler score) and (2) patients preferred to take more convenient and cheap endoscopic treatment based on the economic conditions, the convenience degree of traffic.

### 2.4. Histologic Evaluation and Follow-Up

R0 resection was defined as a lesion with a tumor-free lateral and basal margin; R1 resection was defined as tumor cells being present on the margin. The follow-up was routinely performed at months 1, 3, 6, 12, 18, and 24 after ESTD. The deadline for follow-up was March 2018. The first contrast-enhanced computed tomography (CT) of the chest and abdomen scans was performed at 3 months after ESTD for invasive cancer or at 6 months after ESTD for noninvasive cancer and then performed at 12 months to assess for distant metastasis. Local recurrence was diagnosed when an iodine-unstained area was detected adjacent to an ESTD scar, and cancer cells were histologically verified by a biopsy specimen. If cancer cells were found at the basal resection margin, an additional surgical esophageal resection or esophageal chemoradiotherapy was performed.

### 2.5. Statistical Analysis

The chi-squared test was used for the comparison of categorical variables. Student's *t*-test was used for continuous and normally distributed variables, and the Mann-Whitney *U*-test was used to compare medians if data were not normally distributed. A *p* value of <0.05 was considered statistically significant. Statistical analyses were performed using SPSS Statistics 21 for Windows.

## 3. Results

### 3.1. Patient Characteristics

The baseline characteristics of the included lesions are described in Table 1. Of the 124 patients, the mean observed lesion size was 22.2 ± 11.6 cm^2^ (range, 4.8-83.9 cm^2^). The macroscopic type was 0-IIa in 10 (8.1%) patients, 0-IIb in 28 (22.6%) patients, 0 − IIa + 0 − IIb in 39 (31.4%) patients, 0 − IIb + 0 − IIc in 3 (2.4%) patients, and 0 − IIa + 0 − IIc in 44 (35.5%) patients, according to the Paris classification [6]. ESSTD was performed in a total of 54 (43.5%) patients, two-tunnel ESMTD in 43 (34.7%) patients, three-tunnel ESMTD in 19 (15.3%) patients, and four-tunnel ESMTD in 8 (6.5%) patients. Intramucosal squamous cell cancer was confirmed by pathological examination in 92 (74.2%) patients. Among these patients, 36 (29%) cases of confirmed squamous cell carcinoma had an M1 invasion depth, 27 (21.8%) cases were M2, and 29 (23.4%) cases were M3. Twenty-two (17.7%) were SM1, 1 (0.8%) was low-grade intraepithelial neplasia (LGIN), and 9 (7.3%) were high-grade intraepithelial neoplasia (HGIN) [7].

### 3.2. Procedure Characteristics

In the 124 patients, the mean operative time was 106.3 ± 42.9 min (range, 35-240 min) and the total dissection speed was 22.8 ± 12.7 mm^2^/min (range, 7.1-73 mm^2^/min) (Table 2). There were 41 cases of complete circumferential lesions, 83 cases of noncomplete circumferential lesions, 54 cases of ESSTD and 70 cases of ESMTD. Although the operative time for the complete circumferential lesions was significantly higher than that for the noncomplete circumferential lesions (130.8 ± 44.9 min vs 94.1 ± 36.4 min, *p* ≤ 0.001), there were no significant differences in the dissection speed between the two types of lesion (24.5 ± 13.6 mm^2^/min vs 22.0 ± 12.3 mm^2^/min, *p* = 0.317) (Table 3).

Additional esophagectomy was required for 11 patients due to the depth of infiltration (beyond SM1) or the presence of cancer cells at the basal or lateral resection margin. However, only a single lesion in the resected specimen was found to be invasive (Table 2).

En bloc dissection was achieved in all lesions. The R0 resection rate was 70.2% in our study. There were 53 lesions with positive resection margins, accounting for 42.7% of total cases, which included 15 cases of LGIN, 28 cases of HGIN, and 10 cases of invasive cancer at the lateral or basal cutting edge of the tissue specimen.

The R0 resection rate, R1 resection rate, and the circular muscle damage rate for complete circumferential lesions and noncomplete circumferential lesions were 65.9%, 34.1%, and 48.8%, respectively, and 72.3%, 27.7%, and 48.2%, respectively, with no statistically significant differences between them (*p* = 0.461, 0.461, and 0.951, respectively). The incidence of postoperative esophagus stricture for complete circumferential lesions was significantly higher than that for noncomplete circumferential lesions (82.9% vs 24.1%, *p* ≤ 0.001).

Intraoperative perforation occurred in 1 patient (1.7%). Subcutaneous emphysema and mediastinal emphysema were observed in this patient. This was a noncomplete circumferential lesion that was dissected by ESSTD. No serious immediate or delayed bleeding was found. Although the circular muscle of the muscularis propria was slightly damaged in 60 (48.4%) patients during the operation, no further management was required except for the use of titanium clips in 5 patients. A total of 7 patients (5.6%) suffered from cardiac mucosal laceration and 2 patients (1.6%) experienced gastric fundus perforation (corrected by endoscopic suture); no additional treatment was required for these cases.

All the patients were followed up for 1-44 months. The esophageal stricture rate was 37.1% in our study. The incidence of postoperative stricture was 3.2% (4/124) when the circumferential lesion was 3/4, 6.5% (8/124) when 4/5, 4.8% (6/124) when 5/6, 1.6% (2/124) when 7/8, and 27.4% (34/124) when circumferential lesions were complete. The stenosis rate of complete circumferential lesions was significantly higher than that of the noncomplete circumferential lesions (82.9% vs 24.1%, *p* ≤ 0.001).

Patients with post-ESTD stricture underwent a mean of 5.8 (1-18) esophageal balloon dilatation sessions (Boston Scientific Corp., Marlborough, America) with a diameter of 12-13.5 mm or required a Savary-Gilliard Dilator (Wilson-Cook Medical, America) with the largest diameter being 12.8 mm. Three patients with esophageal stricture underwent fully covered esophageal stent placement (retrievable esophageal stents; Micro-Tech Co., Nanjing, China), with a diameter of 20 mm and a length of 60-120 mm, into the stenosis site to resolve symptoms according to the composite factors from both patients and physicians.

## 4. Discussion

An increasing number of studies have reported a case series of ESTD for the treatment of superficial esophageal squamous cell neoplasms [4, 8–11]. Recent comparative retrospective studies revealed that ESTD is a safe and effective alternative for large esophageal superficial neoplasms, resulting in a shortened operative time, a higher dissection speed, and an increased radical curative rate in comparison with ESD [12, 13]. Although these studies took place in different medical institutions and the procedures were performed by endoscopists of varying technical expertise and degree of training, ESTD appeared to be more effective and safe compared to ESD, which led us to select this procedure for our study.

As far as we know, our study includes the largest sample size of near-circumferential and circumferential superficial esophageal neoplastic lesions dissected by ESTD. The operation pattern involves not only a single tunnel but also multi-tunnel in more than half of the patients. The median procedure time was 106.3 ± 42.9 min and the dissection speed was 22.8 ± 12.7 mm^2^/min. Beyond all doubt, ESTD requires a few more steps and time than ESD in order to create the tunnels; however, once the tunnel has been built, the resection rate can be accelerated by the clearer operative view in the tunnel, saving on the total operating time.

The R0 resection rate was 70.2% in our study; previous studies report an 81.8%-100% resection rate [4, 8–11, 14–18]. We believe that it is more difficult to achieve complete resection (R0) in large and multifocal lesions because the range of dissection spans the adjacent lighter lesion area. The positive resection margins of post-ESD or ESTD specimens are also worthy of consideration. The rate of positive resection margins varies significantly in the literature from 1.7 to 22% [9, 10, 19, 20]. Our results showed that the positive resection margin rate reached 42.7%. We note that there were 37 (29.8%) patients with multifocal lesions in the 53 patients with a positive resection margin. Endoscopists prioritize management of the most serious area with multifocal lesions; one aim is to avoid creating a wound surface too wide or a wound location too high by excessive removal of all multifocal lesions; therefore, the margin of the wound will inevitably pass through the lighter lesion area, which led to a positive resection margin. On the other hand, subsequent treatment decisions, such as an additional esophagectomy or radiofrequency ablation, must be determined by the postoperative pathology of the previously resected specimen. In the 53 patients with positive resection margins, neither surgery nor additional ESD/EMR was required in the 15 cases of LGIN and 28 cases of HGIN; no recurrence was noted after 1-44 months of follow-up. Of the additional 10 patients with positive resection margins, two underwent an esophagectomy and one patient underwent radiotherapy but only one lesion in the resected specimen was found to be invasive cancer. The remaining seven patients in this group refused additional surgery or chemoradiotherapy; no recurrence was noted in these individuals after 4-41 months of follow-up. Over a median follow-up duration of 19.1 ± 12.4 months, 84.9% of patients were found to be free of any neoplasia, suggesting that the rim of coagulation necrosis resulting from ESTD may have eradicated the marginal dysplasia in many of these patients [21].

Recently published studies of ESTD for the treatment of superficial esophageal squamous cell neoplasms [4, 8–11, 14, 16, 17] report rates of immediate bleeding, delayed bleeding, and perforation as 5.6-28.6%, 5.9%, and 4%, respectively. The percentages of bleeding and perforation were higher compared to those of ESD, which were reported as 0-5.2% and 0-6.9%, respectively [20, 22–26]. In our study, the rates of immediate bleeding and perforation were 0 and 0.8%, which were lower than the previously reported results. A primary clip closure was used for endoscopic closure of small damages and minor defects, and no further management was needed. Consequently, our study found that ESSTD or ESMTD exhibited advantages in reducing operation-related complications for esophageal lesions more than three-quarters of the esophageal lumen circumference.

Post-ESD esophageal stricture developed in 12-17% of patients, with risk factors including the circumference and length of the resection and the histological depth [27–30]. ESD resections encompassing more than 75% of the esophageal lumen and the depth of invasion beyond M_2_ are reliable predictors of postoperative stricture [20, 31]. In our study, the incidence of postoperative stricture was 43.5%. A similar study performed by Tang et al. [32] reported a 45% (18/40) rate of post-ESD stricture. We report a significantly higher rate than those previously reported, which is likely to be because the lesions that we assessed involved more than 75% of the circumference of the esophagus. The risk factor for post-ESTD esophageal strictures will be discussed in our next article.

In summary, ESSTD or ESMTD is a time-saving dissection technique for near-circumferential and circumferential esophageal lesions and is associated with fewer long-term complications. Our results showed a high positive margin during this process; however, we considered that positive resection margins in any large and multifocal lesions are inevitable, regardless of the type of endoscopic procedure used. Moreover, no recurrence has been found so far. Therefore, we suggest that there is no need for unlimited extended resection areas for multifocal lesions to reduce the rate of positive resection margins. It is open to discussion whether additional surgery for such patients is required, and additional research is needed to sufficiently address this question. In conclusion, ESTD, especially ESMTD, is recommended for the treatment of large superficial esophageal neoplastic lesions that occupy more than three-quarters of the esophageal circumference, including circumferential superficial esophageal neoplastic lesions.

## Figures and Tables

**Table 1:** Clinicopathological characteristics of the patients.

Number of patients	124
Age, mean ± SD (range) (years)	63.1 ± 7.9 (41-83)
Sex (male, female)	76 (48)
Size, mean (range) (cm^2^)	22.2 ± 11.6 (4.8-83.9)
Circumferential extent (*N*) (%)	
3/4	39 (31.4%)
4/5	25 (20.2%)
5/6	15 (12.1%)
7/8	4 (3.2%)
1	41 (33.1%)
Macroscopic type (*N*) (%)	
0-IIb	28 (22.6%)
0 − IIa + 0 − IIb	39 (31.4%)
0 − IIb + 0 − IIc	3 (2.4%)
0 − IIa + 0 − IIc	44 (35.5%)
0-IIa	10 (8.1%)
Multifocal lesions (*N*) (%)	37 (29.8%)
Number of tunnels (*N*) (%)	
Single tunnel	54 (43.5%)
Two tunnels	43 (34.7%)
Three tunnels	19 (15.3%)
Four tunnels	8 (6.5%)
Depth of invasion (*N*) (%)	
Low-grade intraepithelial neoplasia	1 (0.8%)
High-grade intraepithelial neoplasia	9 (7.3%)
M1	36 (29%)
M2	27 (21.8%)
M3	29 (23.4%)
SM1	8 (6.4%)
>SM1	14 (11.3%)

**Table 2:** Endoscopic features of the lesions.

Operative time, mean (min)	106.3 ± 42.9 (35-240)
Dissection speed, mean (range) (mm^2^/min)	22.8 ± 12.7 (7.1-73)
En bloc resection (%)	124 (100%)
R0 resection	87 (70.2%)
R1 resection	37 (29.8%)
Positive resection margins (*N*) (%)	53 (42.7%)
Follow-up, mean (range) (mon)	19.1 ± 12.4 (1-44)
Additional esophagectomy (*N*) (%)	11 (8.9%)
Residual cancer	1
Complications (*N*) (%)	
Perforation	1 (0.8%)
Damaged of circular muscle	60 (48.4%)
Cardiac mucosal laceration	7 (5.6%)
Gastric fundus perforation patients	2 (1.6%)
Stenosis	54 (43.5%)

**Table 3:** Comparison of endoscopic features between complete circumferential (CC) and noncomplete circumferential lesions (non-CC).

CC (*N* = 41)		Non-CC (*N* = 83)	*p*
Operative time, mean (min)	130.8 ± 44.9	94.1 ± 36.4	≤0.001
Dissection speed, mean (mm^2^/min)	24.5 ± 13.6	22.0 ± 12.3	0.317
Size, mean (cm^2^)	28.9 ± 13.4	18.8 ± 8.9	≤0.001
En bloc resection (%)	41 (100%)	83 (100%)	
R0 resection	27 (65.9%)	60 (72.3%)	0.461
R1 resection	14 (34.1%)	23 (27.7%)	
Complications (*N*) (%)			
Perforation	0	1	
Damage of circular muscle	20 (48.8%)	40 (48.2%)	0.951
Stenosis	34 (82.9%)	20 (24.1%)	≤0.001

## Data Availability

The data used to support the findings of this study are included within the article.

## References

[1] Chinese Society of Digestive Endoscopy (2015). Diagnosis and treatment of early esophageal squamous cell carcinoma and precancerous lesions in China. *Chinese Journal of Digestive Endoscopy*.

[2] Kuwano H., Nishimura Y., Oyama T. (2015). Guidelines for Diagnosis and Treatment of Carcinoma of the Esophagus April 2012 edited by the Japan Esophageal Society. *Esophagus*.

[3] Zhai Y. Q., Li H. K., Linghu E. Q. (2016). Endoscopic submucosal tunnel dissection for large superficial esophageal squamous cell neoplasms. *World Journal of Gastroenterology*.

[4] Linghu E., Feng X., Wang X., Meng J., Du H., Wang H. (2013). Endoscopic submucosal tunnel dissection for large esophageal neoplastic lesions. *Endoscopy*.

[5] Gan T., Yang J.-L., Zhu L.-L., Wang Y.-P., Yang L., Wu J.-C. (2016). Endoscopic submucosal multi-tunnel dissection for circumferential superficial esophageal neoplastic lesions (with videos). *Gastrointestinal Endoscopy*.

[6] Lambert R. (2003). The Paris endoscopic classification of superficial neoplastic lesions: esophagus, stomach, and colon: November 30 to December 1, 2002. *Gastrointestinal Endoscopy*.

[7] Japan Esophageal Society (2017). Japanese classification of esophageal cancer, 11th edition: part I. *Esophagus*.

[8] Pioche M., Mais L., Guillaud O. (2013). Endoscopic submucosal tunnel dissection for large esophageal neoplastic lesions. *Endoscopy*.

[9] Gao X., Shan H., Li Y., Luo G., Xu G. (2012). Application of submucosal tunneling endoscopic resection for early esophageal cancer and precancerous lesions. *Linchuang Waike Zazhi*.

[10] Arantes V., Albuquerque W., Freitas Dias C. A., Demas Alvares Cabral M. M., Yamamoto H. (2013). Standardized endoscopic submucosal tunnel dissection for management of early esophageal tumors (with video). *Gastrointestinal Endoscopy*.

[11] Xiong Y., Li Y., Yuan H., Geng Y., Zhang Z., Wang A. (2013). The application of endoscopic submucosal tunnel dissection (ESTD) in treating early esophageal cancer and precancerous lesions. *Linchuang Xiaohuabing Zazhi*.

[12] Zhai Y., Linghu E., Li H. (2014). Comparison of endoscopic submucosal tunnel dissection with endoscopic submucosal dissection for large esophageal superficial neoplasms. *Nan Fang Yi Ke Da Xue Xue Bao*.

[13] Huang R., Cai H., Zhao X. (2017). Efficacy and safety of endoscopic submucosal tunnel dissection for superficial esophageal squamous cell carcinoma: a propensity score matching analysis. *Gastrointestinal Endoscopy*.

[14] Linghu E., Li H., Huang Q. (2011). Using tunnel technology dissecting long cirucumferencial lesions of esophagus. *China Continuing Medical Education*.

[15] Zhai Y., Linghu E., Li H. (2014). Double-tunnel endoscopic submucosal tunnel dissection for circumferential superficial esophageal neoplasms. *Endoscopy*.

[16] Tan Y., Li C., Liu D., Huo J. (2014). Endoscopic submucosal tunnel dissection for one large high-grade intraepithelial neoplasia. *Chinese Journal of Digestive Endoscopy*.

[17] Zhou Z., Huang Z., Cheng H., Dai X., Li Y., Tang J. (2014). Endoscopic submucosal tunnel dissection for large early esophageal cancers and precancerous lesions. *Chinese Journal of Digestive Endoscopy*.

[18] Park J. S., Youn Y. H., Park J. J., Kim J. H., Park H. (2016). Clinical outcomes of endoscopic submucosal dissection for Superficial esophageal squamous neoplasms. *Clinical Endoscopy*.

[19] Repici A., Hassan C., Carlino A. (2010). Endoscopic submucosal dissection in patients with early esophageal squamous cell carcinoma: results from a prospective Western series. *Gastrointestinal Endoscopy*.

[20] Ono S., Fujishiro M., Niimi K. (2009). Long-term outcomes of endoscopic submucosal dissection for superficial esophageal squamous cell neoplasms. *Gastrointestinal Endoscopy*.

[21] ASGE Technology Committee, Maple J. T., Abu Dayyeh B. K. (2015). Endoscopic submucosal dissection. *Gastrointestinal Endoscopy*.

[22] Isomoto H., Yamaguchi N., Minami H., Nakao K. (2013). Management of complications associated with endoscopic submucosal dissection/endoscopic mucosal resection for esophageal cancer. *Digestive Endoscopy*.

[23] Honda K., Akiho H. (2012). Endoscopic submucosal dissection for superficial esophageal squamous cell neoplasms. *World Journal of Gastrointestinal Pathophysiology*.

[24] Sun F., Yuan P., Chen T., Hu J. (2014). Efficacy and complication of endoscopic submucosal dissection for superficial esophageal carcinoma: a systematic review and meta-analysis. *Journal of Cardiothoracic Surgery*.

[25] Oyama T., Tomori A., Hotta K. (2005). Endoscopic submucosal dissection of early esophageal cancer. *Clinical Gastroenterology and Hepatology*.

[26] Sohara N., Hagiwara S., Arai R., Iizuka H., Onozato Y., Kakizaki S. (2013). Can endoscopic submucosal dissection be safely performed in a smaller specialized clinic?. *World Journal of Gastroenterology*.

[27] Takahashi H., Arimura Y., Masao H. (2010). Endoscopic submucosal dissection is superior to conventional endoscopic resection as a curative treatment for early squamous cell carcinoma of the esophagus (with video). *Gastrointestinal Endoscopy*.

[28] Mizuta H., Nishimori I., Kuratani Y., Higashidani Y., Kohsaki T., Onishi S. (2009). Predictive factors for esophageal stenosis after endoscopic submucosal dissection for superficial esophageal cancer. *Diseases of the Esophagus*.

[29] Ono S., Fujishiro M., Niimi K. (2009). Predictors of postoperative stricture after esophageal endoscopic submucosal dissection for superficial squamous cell neoplasms. *Endoscopy*.

[30] Kim J. S., Kim B. W., Shin I. S. (2014). Efficacy and safety of endoscopic submucosal dissection for superficial squamous esophageal neoplasia: a meta-analysis. *Digestive Diseases and Sciences*.

[31] Shi Q., Ju H., Yao L. Q. (2014). Risk factors for postoperative stricture after endoscopic submucosal dissection for superficial esophageal carcinoma. *Endoscopy*.

[32] Tang B., Bai J. Y., Zhao X. Y. (2014). Endoscopic submucosal dissection for superficial esophageal cancer with near-circumferential lesions: our experience with 40 patients. *Surgical Endoscopy*.

